# Traditional and Emerging Sex-Specific Risk Factors for Cardiovascular Disease in Women

**DOI:** 10.31083/j.rcm2308288

**Published:** 2022-08-16

**Authors:** Puja K. Mehta, Scott Gaignard, Arielle Schwartz, JoAnn E. Manson

**Affiliations:** ^1^Emory Women’s Heart Center and Emory Clinical Cardiovascular Research Institute, Division of Cardiology, Emory University School of Medicine, Atlanta, GA 30322, USA; ^2^J. Willis Hurst Internal Medicine Residency Program, Emory University, Atlanta, GA 30322, USA; ^3^Division of Preventive Medicine, Brigham and Women’s Hospital, Harvard Medical School, Boston, MA 02215, USA

**Keywords:** heart disease, sex-specific risk factors, women

## Abstract

Cardiovascular disease (CVD) remains a major health threat in women. While traditional CVD risk factors such as hypertension, hyperlipidemia, diabetes, and smoking have been recognized for over 50 years, optimal control of these risk factors remains a major challenge. Unique sex-specific risk factors such as adverse pregnancy outcomes, premature menopause and low estrogen states, and chronic autoimmune inflammatory disorders also contribute to increased CVD risk in women. In addition, psychological risk factors such as stress, depression, and social determinants of health may have a disproportionately adverse impact in women. An improved understanding of traditional and emerging sex-specific CVD risk factors and management of modifiable factors is critical for clinicians who provide care for women. Early recognition and treatment of risk factors may alter the trajectory of adverse CVD events. A multi-disciplinary approach with team-based care involving multiple specialists and improved, targeted educational efforts are needed to reduce CVD risk factors and its adverse consequences in women.

## 1. Introduction 

**Fig. 1.**
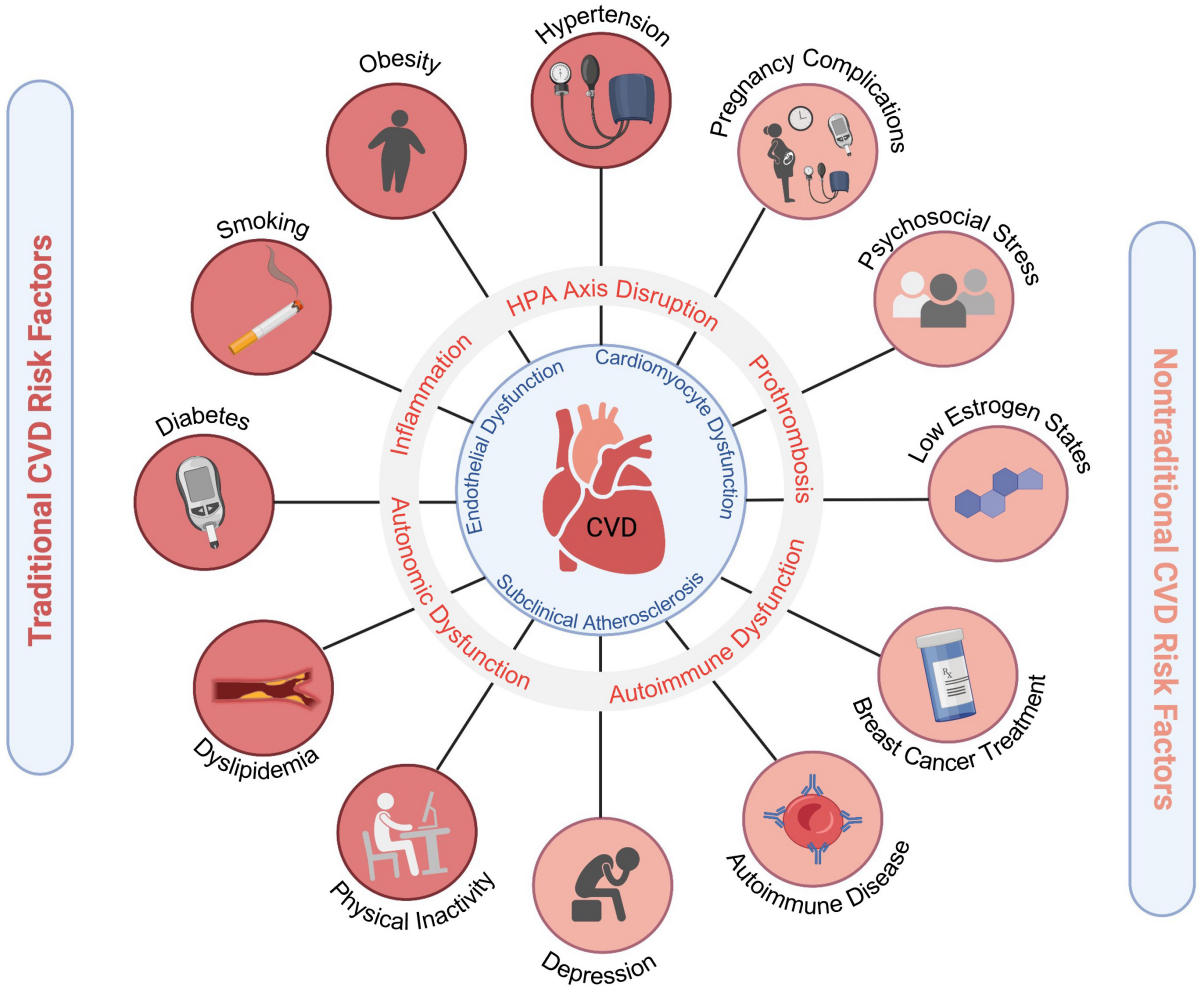
**Cardiovascular risk factors in women**. Traditional and emerging risk factors contribute to pro-atherosclerotic, pro-inflammatory, pro-thrombotic states that ultimately lead to increased CVD morbidity and mortality in women. Adverse pregnancy outcomes include hypertensive disorders of pregnancy, gestational diabetes, and pre-term birth. Figure created using https://Biorender.com.

Among cardiovascular diseases (CVD), ischemic heart disease remains the leading cause of morbidity and mortality in both men and women, followed by stroke [1]. While ischemic heart disease typically presents a decade later in women compared to men, women have less favorable outcomes with higher mortality, and increased angina, and health-related morbidities [2, 3]. This disparity is in part due to increased comorbid risk factors in women, delays in presentation and treatment, and less use of guideline-based therapies [2, 4]. Women have higher rates of traditional risk factors such as hypertension (HTN), diabetes, and obesity. In addition to the traditional CVD risk factors that are common in both men and women, there are also sex-specific risk factors that are either more prevalent or unique to women. These factors contribute to CVD risk via mechanisms of inflammation, autonomic dysregulation, and hypothalamic-pituitary-adrenal (HPA) hormonal axis disruption. This results in a pro-atherogenic and pro-thrombotic milieu which increases the incidence of myocardial infarction, heart failure, stroke, and CVD death (Fig. 1). Over the past three decades it has also become increasingly apparent that there are sex differences in the pathophysiology of heart disease [5]. For example, there are certain conditions that predominate in women such as ischemia with no obstructive coronary artery disease (INOCA), myocardial infarction with no obstructive coronary artery disease (MINOCA), spontaneous coronary artery dissection (SCAD), Takotsubo syndrome, and heart failure with preserved ejection fraction (HFpEF). Mortality from heart disease among young women (less than age 55 years) is increasing and is associated with more risk factors and comorbidities [6, 7, 8].

A summary of updated recommendations for primary prevention of CVD in women was recently published [9]. In primary prevention, CVD risk is estimated based on various risk scores such as the ACC/AHA ASCVD pooled cohort equation risk calculator, Framingham risk score, Reynold’s risk score, and others, but none of these tools incorporate emerging/novel sex-specific markers. Whether incorporation of these novel risk factors would improve CVD risk prediction remains uncertain. Coronary artery calcium scoring is another powerful tool that can be helpful in borderline risk patients to help determine how aggressive to target primary prevention strategies such as statins [10]. In order to reduce CVD-related morbidity and mortality, it is imperative to improve access to healthcare, identify CVD risk factors earlier, promote heart-healthy lifestyle choices, and implement pharmacologic treatment expeditiously when indicated. This review highlights traditional (Table 1, Ref. [11]) and emerging risk factors for CVD that can be treated to reduce morbidity and mortality in women.

**Table 1.** **Traditional cardiovascular risk factors**.

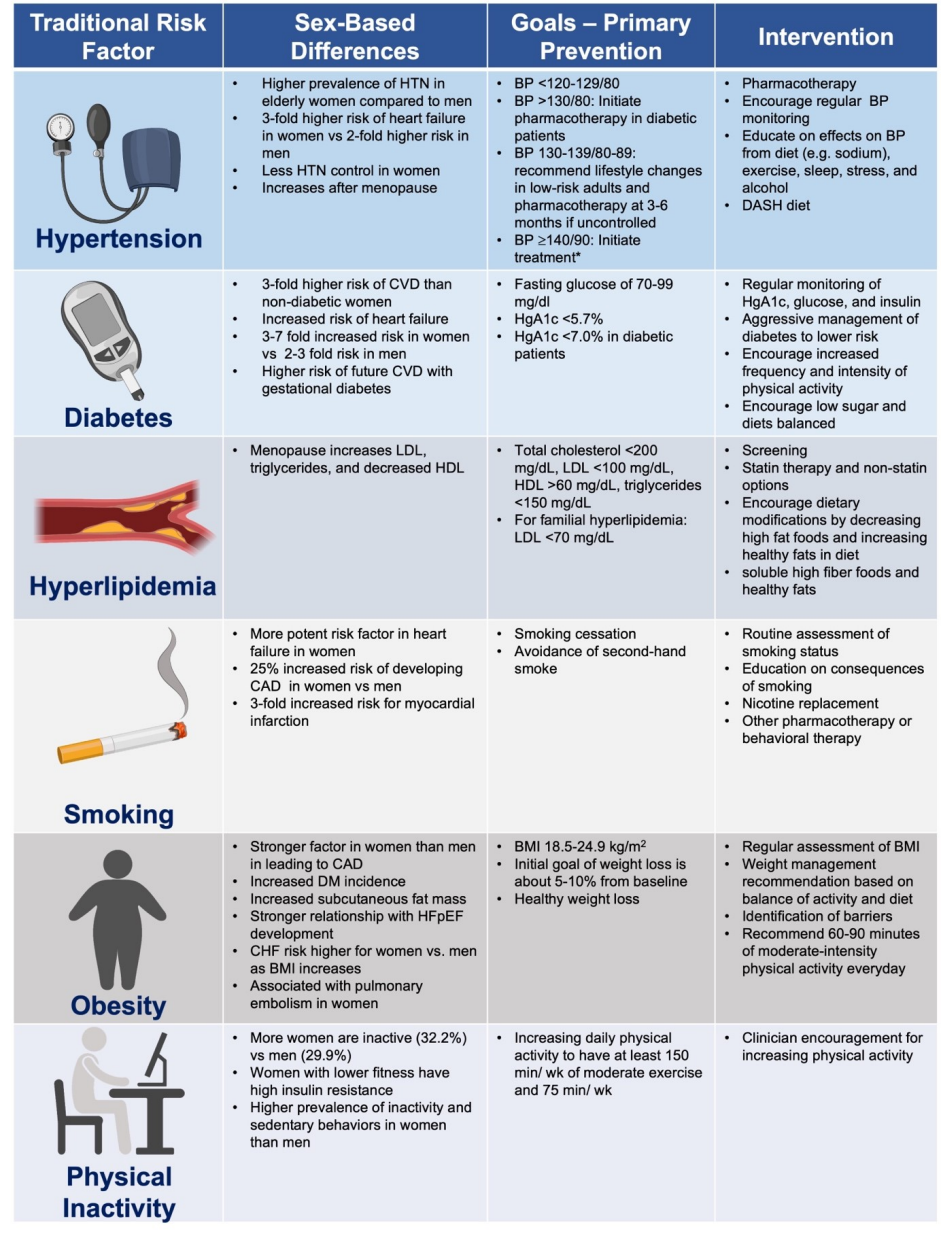

*AHA recommendations for management of stage 1 hypertension in low-risk adults [11]. Family history of premature coronary heart disease is a risk factor that should also be considered in decision-making.

## 2. Traditional CVD Risk Factors in Women 

### 2.1 Hypertension (HTN)

HTN is a highly prevalent and powerful CVD risk factor in both women and men that is associated with myocardial infarction, heart failure, stroke, atrial fibrillation, peripheral vascular disease, and chronic kidney disease. Systolic blood pressure begins to increase around the time of transition to menopause in women and becomes more prevalent after 65 years of age in women compared to men. In addition, studies have found that the risk of HTN is higher in women than men [12, 13, 14]. Young women with HTN are at greater risk of end-organ damage compared to age-matched men [15]. Arterial stiffness, autonomic dysfunction, generalized endothelial dysfunction, and upregulation of the renin-angiotensin system all contribute to these increases in blood pressure in the setting of declining estrogen levels [16]. African American (AA) women are more likely to have HTN diagnosed at younger ages and have more severe HTN compared to white women [17]. In addition, AA women have nearly twice the age-adjusted death rate attributable to HTN, although this and other CVD risk factors are often undertreated in AA women. A recent community-based CVD risk factor screening study in 945 AA women found that elevated blood pressure and obesity were prevalent at younger ages [18]. Older and AA women are particularly susceptible to increased risk from heart failure, stroke, and renal disease due to HTN [19]. Women are also more likely to have anxiety-related and “white coat” HTN compared to men [20, 21]. Current HTN guidelines recommend avoiding the use of oral contraceptives in reproductive-age women with uncontrolled HTN seeking contraception and instead recommend using low dose ethinyl estradiol, progestin only formulation, or alternative methods such as an intrauterine device [14].

### 2.2 Diabetes

Diabetes mellitus remains one of the most prevalent diseases in the United States with an estimated one in nine women having this condition [22, 23]. The inflammation and atherogenesis associated with diabetes results in an increased risk of CVD [24, 25, 26]. Diabetic women have a 3–7 times increased risk of developing heart disease compared to a 2–3 times increased risk in diabetic men [27, 28, 29, 30]. The greater impact of diabetes on women may be partially due to increased adiposity [31]. Women are more likely than men to progress from prediabetes to diabetes [32]. In the original cohort of the Framingham Heart Study, there was a strong, independent association between HgA1c and CVD in women, but not in men. For every 1% increase in HgA1c, the relative odds for CVD increased by 1.39 in women [33]. The strong association in women between hyperglycemia and CVD remained significant even when women who were classified as diabetics were excluded [33]. In a meta-analysis, the rate of fatal coronary heart disease was higher among patients with diabetes compared to those without diabetes (5.4% vs. 1.6%) [27]. However, the difference was more pronounced in diabetic vs. non-diabetic women (7.7% vs. 1.2%) compared to diabetic vs. non-diabetic men (4.5% vs. 2.0%) [27]. Women with diabetes had a higher relative risk of fatal coronary heart disease compared to diabetic men (3.5 vs. 2.0 respectively) [27]. Menopause results in additional risk factors for CVD [27]. Although there does not seem to be a correlation between the onset of menopause and higher glucose levels, an increased risk of metabolic syndrome emerges after the transition to menopause, beyond the risk attributable to age alone [34, 35, 36]. Early diagnosis of diabetes is essential, particularly in racial/ethnic groups at high risk for diabetes such as AA, Hispanics, American Indians, and Pacific Islander Americans. While glucose-lowering therapy reduces microvascular complications, randomized trials have not shown a benefit of intensive glucose lowering on CVD outcomes in patients with long-standing diabetes [37, 38].

### 2.3 Dyslipidemia 

Hyperlipidemia is a well-established modifiable risk factor for CVD, with a direct relationship between low-density lipoprotein (LDL) cholesterol and ASCVD events. Studies have shown that lowering LDL is associated with decreased risk for CVD in both primary and secondary prevention populations which have included women [39]. The INTERHEART trial was a large case control study that sought to quantify the risk of various modifiable risk factors for CVD. Smoking and an abnormal lipid profile (defined as an elevated ApoB/ApoA1 ratio) were the two strongest risk factors for myocardial infarction, with abnormal lipid profiles being the highest of all the risk factors studied [40]. The 2013 ACC/AHA Guideline on the Treatment of Blood Cholesterol recommends initiation of statins in both women and men with an LDL-C >190 mg/dL or an estimated 10-year ASCVD risk of ≥7.5% based on the pooled cohort equation risk calculator, which incorporates sex in the calculation [41]. Statin therapy has similar benefits for both women and men in CVD risk reduction, although elderly women are 20% less likely than men to use statins, which may be due to either under prescription or to the higher prevalence of statin-associated myalgias [42, 43, 44].

Cholesterol levels may fluctuate throughout a woman’s life-span, from young adult to pregnancy to the transition to menopause, and treatment should be tailored accordingly. Women are at increased risk of hyperlipidemia, with the postmenopause period being a particularly vulnerable time [45]. Following menopause, women have higher total cholesterol, triglycerides, and LDL, and reduced HDL, which places them at higher risk for CVD [46]. High non-HDL-C and triglyceride levels are more important CVD risk factors in women than men, especially women with diabetes [47, 48].

An elevated lipoprotein(a) level is considered a risk factor that is pro-thrombotic, pro-inflammatory, and pro-atherogenic in both men and women and is associated with a higher risk of CVD events [39]. In the Multi-Ethnic Study of Atherosclerosis (MESA), Lp(a) was associated with increased CVD risk when there was evidence of systemic inflammation as determined by higher C-reactive protein levels (≥2 mg/L) [49]. A randomized, placebo-controlled trial of the impact of lowering Lp(a) on major CVD events is currently ongoing (NCT04023552) [50]. While there are no pharmacologic agents available for routine clinical use to lower Lp(a) levels, small interfering RNA (siRNA) agents that directly inhibit Lp(a) messenger RNA translation in the liver are promising [51]. A review of the management of blood cholesterol throughout a woman’s life cycle, ranging from pre-pregnancy, pregnancy, pre- and perimenopause, postmenopause, and at older ages has recently been published [52]. Table 2 (Ref. [39, 53]) summarizes the current recommendations for the use of therapies such as statins and aspirin for primary and secondary prevention of CVD in women.

**Table 2.** **Recommendations for ASCVD secondary and primary prevention in women**.

Recommendations for ASCVD Secondary and Primary Prevention in Women		
	YES	NO
${\mathrm{Aspirin}}^{*}$	∙ Coronary heart disease (secondary prevention)	∙ Healthy women with no major CVD risk enhancing factors
	∙ Prior TIA/ stroke (secondary prevention)	∙ Older age (>60)***
	∙ Peripheral artery disease (secondary prevention)	∙ History of bleeding or high bleeding risk
Statins^**^	∙ Clinical ASCVD (secondary prevention)	∙ Age 40 to 75 years with low risk (≤5% CVD risk)
	∙ Ages 40 to 75 years with intermediate risk (≥7.5% to <20% CVD risk + risk enhancing ${\mathrm{factors}}^{\#}$)	∙ Pregnant/Planning pregnancy
	∙ Age 40 to 75 years with high risk (≥20% CVD risk)	
	∙ Familial hyperlipidemia (LDL-C ≥190 mg/dL)	
	∙ Diabetes mellitus	

* Clinician-patient discussion to consider aspirin if low risk for bleeding and high ASCVD risk in those 40 to 59 (≥10% 10-year CVD risk) (C recommendation) [53]. Consider family history of premature coronary heart disease in decision-making. ** Could consider statin if at age 40 to 75 years with borderline risk (5% to ≤7.5% CVD risk + risk enhancing factors). *** USPSTF Recommendations [53]. # ACC/AHA Blood Cholesterol Guidelines [39].

### 2.4 Smoking

Cigarette smoking is the leading behavioral contributor for CVD mortality, with an estimated 30.8 million adults in the United States currently smoking cigarettes [54]. It is associated with multiple forms of CVD, including coronary heart disease, cerebrovascular disease, and peripheral arterial disease [55]. While men are more likely to smoke cigarettes (14.1% vs. 11%), women are disproportionately affected by the risk of CVD [54, 56]. The surgeon general in a weekly morbidity and mortality report in 2002 wrote, “Like their male counterparts who smoke, women smokers are at increased risk of cancer, cardiovascular disease, and pulmonary disease, but women also experience unique risks related to menstrual and reproductive function…it is tragic that an entirely preventable factor continues to claim so many women’s lives” [57]. Huxley *et al*. [56] conducted a meta-analysis of cohort studies that included 2.4 million individuals and found that compared to non-smokers, women who smoke have a 25% greater relative risk of coronary heart disease than male smokers, after adjusting for other risk factors. All patients should be asked about tobacco use at each office visit; and smokers should be advised to quit and given the tools to do so [58]. Furthermore, AHA/ACCF guidelines also recommend that patients should be advised to avoid environmental tobacco exposure [59].

Electronic cigarettes (e-cigarettes) have been growing in popularity since their introduction in 2007 and contain hazardous compounds [60]. One study found that within one month of switching from tobacco smoking to e-cigarettes, endothelial function assessed by flow mediated dilation (FMD) and vascular stiffness (measured by pulse wave velocity) improved in both men and women, but women had more improvement compared to men [61]. However, other studies have found impaired vascular function with both tobacco cigarettes and e-cigarettes [60]. In a retrospective study of 96,000 participants, those who smoked e-cigarettes were more likely to have myocardial infarction (odds ratio: 1.56) and stroke (odds ratio: 1.3) compared to non-e-cigarette users [62]. Whether e-cigarettes pose a relatively higher risk in women compared to men has not been studied. In a study with over 400 pregnant women, 6.5% were using e-cigarettes during pregnancy and 65% stated that e-cigarettes were safer than tobacco cigarettes. Future studies are needed to address the safety of e-cigarettes on women and fetuses [63].

### 2.5 Obesity

An elevated body mass index (BMI) is associated with increased CVD risk in both women and men, but there are important sex differences in fat distribution (visceral vs. subcutaneous) [64]. Women predominantly accumulate subcutaneous fat, while men accumulate more visceral fat. However, with menopause, women have increased visceral fat compared to premenopausal women of similar ages, which contributes to insulin resistance and inflammation [65]. Cardiometabolic biomarkers such as serum adiponectin levels also predict CVD risk and mortality in both men and women [66, 67]. Several CVD risk factors are increased in patients with greater adiposity and obesity. For example, HTN is associated with overweight (OR = 2.1) and obesity (OR = 5.2) in women, and diabetes is associated with abdominal obesity (OR = 3.9) in women [68]. Weight reduction is associated with improvements in cholesterol levels, lower blood pressure, and a lower risk of developing type 2 diabetes [69]. Overweight and obese individuals and women with waist circumference of >35 inches (88 cm) should be counseled regarding their elevated CVD risk and referred for nutrition counseling as well as prescribed a diet that leads to a 500–750 kcal/day energy deficit [70]. Given the importance of weight management in CVD, strategies such as bariatric surgery should be considered in women with a BMI ≥40 or in those with BMI ≥35 with comorbid conditions related to obesity, and has been shown to be effective in improving diabetes and other comorbidities [70]. Glucagon-like peptide (GLP-1) agents are a novel class of agents that are associated with weight reduction, decreased insulin resistance, anti-inflammatory effects, and CVD benefits [71].

### 2.6 Physical Inactivity 

Physical activity is one of the most important modifiable risk factors for CVD. In one study, the risk of heart disease with physical inactivity was higher compared to other traditional CVD risk factors [72]. The AHA defines adequate physical activity as 150 minutes/week of moderate intensity or 75 minutes/week of vigorous intensity exercise. Physical activity is also part of the AHA’s Life’s Simple 7, created in 2010, which outlines and defines modifiable risk factors that contribute to cardiovascular health [73]. Physical activity levels are lower among women compared to men, especially in AA and Hispanic adults [13]. Women undergo several events throughout their lives that reduce their amount of physical activity compared to men, including pregnancy and parenting [74]. A recent study analyzing the lifetime risk of coronary heart disease based on genetic factors and/or lifestyle modifications (defined in accordance with AHA’s Life’s Simple 7) of nearly 10,000 participants found that lifestyle factors affect overall freedom from coronary heart disease more than genetic factors [75]. Lifestyle factors also have a greater effect on women than men for future CVD events [75]. In a Swedish cohort study, total physical activity time was inversely associated with a risk of myocardial infarction only in women [76]. There may be a dose-dependent risk of CVD in older women (≥63 years old) based on the degree of sedentary lifestyle [77]. Overall, these recent findings further emphasize the importance of physical activity counseling for women regarding their cardiovascular health, especially with aging and through key transition periods in their lives.

Exercise training leads to direct improvements in vascular function, diastolic function, and beneficially alters autonomic tone [78, 79, 80, 81, 82, 83, 84]. In a meta-analysis of 48 randomized trials of cardiac rehabilitation (CR) vs. usual care in ischemic heart disease patients, CR was associated with reduced cardiac mortality (OR = 0.74; 95% CI: 0.61 to 0.96) and all-cause mortality (odds ratio = 0.80; 95% CI: 0.68 to 0.93) [85]. Despite its beneficial effects on morbidity, mortality, functional capacity, and quality of life, CR is unfortunately grossly underutilized in women [86, 87, 88]. A comprehensive cardiac rehabilitation program includes not only aerobic and strength training exercises, but also nutrition counseling, education on tobacco cessation strategies, and psychological evaluation [89].

### 2.7 Family History

Eliciting a family history of CVD is important, as this risk factor increases CVD risk. A family history of premature CVD in a first-degree relative (defined as <55 years in men and <65 years in women) is considered a risk enhancing factor, although it is not incorporated in the ACC/AHA Atherosclerotic CVD Risk Estimator [10, 90]. In a large study of patients with acute myocardial infarction, a family history of premature coronary heart disease was an independent predictor of major adverse events and cardiac death, especially in women compared to men [91]. In a study combining cohorts from the Physician’s Health Study (n = 22,071 men) and Women’s Health Study (n = 39,876 women), a history of a maternal MI was associated with increased CVD risk in both sons and daughters. However, a history of paternal MI conferred increased risk for sons, but only premature paternal MI (<50 years) conferred increased risk for daughters [92]. In the INTERHEART study, both a paternal and maternal history of MI (defined as having MI at age <50 years) was associated with an increased risk for an MI. The odds ratio for an MI with a paternal history of MI was 1.84 (95% CI: 1.69 to 2.0) vs. 1.72 for a maternal history (95% CI: 1.56 to 1.91) (*p* = 0.692 for heterogeneity) [93].

## 3. Emerging CVD Risk Factors in Women 

### 3.1 Pregnancy-Related Disorders 

Hypertensive disorders of pregnancy are a major cause of maternal morbidity and mortality [94]. Women with pre-eclampsia have not only an increased risk but also an earlier onset of CVD risk factors including HTN, diabetes, and hyperlipidemia [95]. A 2021 Swedish cohort study with over 2 million women and 4 million pregnancies tracked CVD after pre-eclampsia pregnancy complications. CVD mortality rates were higher among those with pre-eclampsia or eclampsia at a hazard ratio (HR) of 2.10, which was the third highest predictor, after stillbirth (3.14) and gestational diabetes (3.03) [96]. The elevated rate of CVD mortality for women with a history of pre-eclampsia was especially pronounced in the 4th post-partum decade [97]. In addition, other adverse pregnancy outcomes (APOs) such as gestational diabetes, preterm birth (<37 weeks gestational age), and small for gestational age were also predictive of future CVD compared to women who did not experience APOs. A large systematic meta-analysis of over 300,000 women with a history of preterm delivery found a 1.4- to 2-fold increase for the risk of future maternal CVD (RR: 1.43), CVD death (1.78), coronary heart disease (1.20), and stroke (1.65), with the highest risks when preterm deliveries occurred <32 weeks of gestation or when medically indicated [95, 98]. These risks were higher in those women who had a greater number of preterm births [98]. Of note, excessive gestational weight gain or persistent weight gain post pregnancy also contributes to risk factors such as HTN and increases future CVD risk [99, 100, 101, 102].

Though the pathogenesis is under active investigation, there appears to be an element of chronic inflammation after a complicated pregnancy, resulting in a long-term increase in CVD risk factors [103, 104]. Although pregnancy complications are established CVD risk predictors, knowledge gaps remain as to how these factors should be incorporated to inform decision-making regarding CVD preventive strategies. Women who have experienced an APO should be routinely screened for CVD risk factors, including HTN, diabetes, hyperlipidemia, smoking cessation, and obesity, with counseling and pharmacotherapy when appropriate. It is recommended that women with adverse pregnancy outcomes undergo CVD risk screening within 3 months post-partum [9].

### 3.2 Hormonal Factors and Conditions 

#### 3.2.1 Menopause 

Endogenous estrogen generally has favorable effects on the vasculature in premenopausal women and is anti-inflammatory, anti-thrombotic, and athero-protective. Declining estrogen levels during the transition to menopause and a shift in the ratio of estrogen/testosterone levels contributes to endothelial dysfunction and vascular aging, resulting in an increased incidence of CVD in women after menopause [105]. Premature menopause (menopause before age 40, either natural or surgical) is a risk factor for early CVD [106, 107]. CVD risk factors such as HTN, hyperlipidemia, and weight gain also become more prevalent after menopause. There is a shift in the lipid profile resulting in an increase in more atherogenic lipoproteins such as LDL and triglycerides and a lowering of the protective HDL. However, while prior observational studies indicated that hormone therapy during menopause may have some benefit, the two large, randomized, controlled Women’s Health Initiative (WHI) trials (conducted in women with a mean age of 63) showed that hormone therapy (0.625 mg conjugated equine estrogen + 2.5 mg medroxyprogesterone acetate) vs. placebo was associated with a higher risk of CVD events compared to placebo in the overall group with an intact uterus [108, 109]. In the WHI-E-alone trial, 0.625 mg of conjugated equine estrogen was compared to placebo in those women without a uterus, and showed no benefit in the reduction of CVD [108]. In stratified analyses by age and time since menopause, results for younger women were generally more favorable than for older women, providing support for the “timing hypothesis” [110, 111, 112]. Moreover, the ELITE trial, which directly tested the timing hypothesis, found that oral estradiol (1 mg/day), with or without progesterone vaginal gel, was associated with less progression of carotid-intima media thickness (CIMT) at a 5-year median follow-up if initiated early (within 6 years postmenopause) compared to placebo [113]. However, in those women who were ≥10 years postmenopause, there was no difference in rate of CMIT progression between estradiol and placebo [113]. While there were differences based on timing of menopause in subclinical atherosclerosis measured by CMIT, there were no differences in coronary artery calcium, stenosis, or plaque quantification among the two groups stratified by timing of menopause [113]. However, in the Kronos Early Estrogen Prevention (KEEPS) Study, use of hormone therapy reduced vasomotor symptoms but had neutral results for coronary artery calcification or carotid intima-medial thickness after 4 years of follow up [114, 115]. A meta-analysis of randomized trials including more than 40,000 postmenopausal women reported that women who started hormone therapy less than 10 years after the menopause had a lower incidence of coronary heart disease, but an increased risk of venous thromboembolism, compared to placebo or no treatment [116]. Given the complex pattern of benefits and risks of menopausal hormone therapy, most guidelines support its use to treat moderate-to-severe vasomotor symptoms but not for primary or secondary prevention of CVD. While there have been some studies indicating that estrogen supplementation may be helpful in women with persistent angina who have coronary vascular dysfunction with no obstructive coronary artery disease, there are currently no randomized trials to support this therapy in this population. Currently, the North American Menopause Society recommends that hormone therapy may be initiated for the treatment of menopausal symptoms in women younger than 60 years of age or are within 10 years of menopause onset but should be avoided in women in late menopause [117, 118]. It is also recommended for the management of premature menopause, irrespective of symptoms. Additional research on transdermal formulations of estrogen, which is less likely to pose thromboembolic risks, has been encouraged. In women with a history of CVD where hormone therapy is contraindicated, non-hormonal treatment options are available for women with vasomotor menopausal symptoms, including selective serotonin reuptake inhibitors, serotonin norepinephrine reuptake inhibitors (SNRI), nutritional modifications, improved sleep hygiene, and other strategies [119]. If an SNRI is initiated, blood pressure should be monitored closely as these agents can contribute to HTN.

Bilateral oophorectomy (BSO) induces a “surgical menopause” and is performed for several indications including risk reduction for women at increased risk of hereditary cancer syndromes such as BRCA gene carriers [120]. Observational studies have reported on the association of BSO and increased CVD risk. The Nurses’ Health Study found an increased risk of coronary heart disease in women with hysterectomy + BSO compared to those with hysterectomy and ovarian conservation, with the risk elevated in those undergoing BSO at ages <45 years [121]. However, in the WHI, in post-menopausal women ages 50–79 who had a history of hysterectomy + BSO (n = 14,254) compared to those with hysterectomy alone (n = 11,194), BSO was not associated with an increased risk of coronary heart disease (HR: 1), stroke (HR: 1.04), CVD (HR: 0.99), or death (HR: 0.98) [122]. In a cohort study of 2094 women, having a hysterectomy alone (even without oophorectomy) was associated with increased cardiometabolic risk factors (HTN, hyperlipidemia, and obesity) and an increased risk of CAD and heart failure, particularly in women with hysterectomy at ages ≤35 years [123].

#### 3.2.2 Polycystic Ovary Syndrome (PCOS)

PCOS is a common endocrine disorder in young women, that is associated with hyperandrogenism, ovulatory dysfunction, and insulin resistance. Cardiac risk factors such as HTN, diabetes, and obesity are prevalent in women with PCOS, and it is recommended that women with PCOS are regularly screened for CVD risk factors. Weight management and regular physical activity may improve their risk profile. Women with PCOS had a higher coronary artery calcium score compared to controls with normal ovulatory cycles [124].

### 3.3 Inflammation

Atherosclerosis is a pathologic, inflammatory process in the vascular wall that is triggered by risk factors such as diabetes, dyslipidemia, and smoking, but also may accelerate due to immune dysregulation from chronic infections such as human immunodeficiency virus (HIV) [125, 126, 127, 128, 129, 130]. Sex hormones play an important role in adaptive and innate immune responses and systemic inflammation in women. Moran *et al*. [130] published a review of mechanisms involving immune dysregulation which contribute to inflammation and increase the risk of CVD in women.

C-Reactive Protein (CRP) is a marker of inflammation and has been shown to be a risk factor for cardiovascular events. It is incorporated in the Reynolds Risk Score for improved risk prediction in women [131]. Inflammatory cytokines are thought to stimulate the liver to produce CRP, which is an acute phase reactant [132]. Elevated high sensitivity CRP (hsCRP) levels are predictive of CVD events [133, 134]. Statins, a mainstay of CVD management and prevention, have been shown to reduce hsCRP levels [135, 136]. Thus the benefit of statins in reducing cardiovascular risk is thought to be in part due to their anti-inflammatory properties. Modulating the inflammatory response to reduce CVD risk has been studied in several clinical trials using agents such as canakinumab and colchicine [137, 138, 139]. The Canakinumab Anti-Inflammatory Thrombosis Outcomes Study (CANTOS) demonstrated that in patients with a history of an MI and high sensitively CRP >2 mg/mL, targeting interleukin-1β (an inflammatory signaling molecule) reduces CVD events compared to placebo [140]. Canakinumab at a dose of 150 mg every 3 months led to significantly lower rates of recurrent CVD events, independent of lipid lowering, suggesting that targeting inflammation plays a role in CVD reduction [140]. Tumor necrosis factor-alpha (TNF-α) therapy, in conditions such as rheumatoid arthritis, may also be associated with improved outcomes in CVD [141].

#### 3.3.1 Rheumatologic and Autoimmune Disorders

Rheumatologic and autoimmune disorders, such as systemic lupus erythematosus (SLE), rheumatoid arthritis, psoriasis, and systemic sclerosis are more prevalent in women, and are associated with increased CVD, including CAD, valvular heart disease, arrhythmias, and pericardial disease [6, 9]. Ischemic heart disease is a leading cause of morbidity and mortality in SLE patients, and AA women are much more likely to be diagnosed with SLE compared to white women. Similarly, rheumatoid arthritis is associated with a two-to-threefold higher risk of ischemic heart disease [142]. This increased risk is attributed to not only an augmented systemic inflammatory response leading to endothelial dysfunction and rupture of vulnerable plaques, but also to microvascular dysfunction [143, 144]. Treatment for autoimmune disorders with corticosteroids may also increase CVD risk, due to weight gain, the development of the metabolic syndrome, and premature atherosclerosis [145]. Coronary artery calcium scores may be more predictive of CVD risk than Framingham risk scores in women with SLE and rheumatoid arthritis [146].

### 3.4 Psychological Stress and Depression

In addition to the above risk factors, several studies have shown that psychological risk factors such as anxiety, work and marital stress, depression, low socioeconomic status and loneliness, are associated with CVD [147, 148, 149, 150, 151, 152, 153, 154]. In the Stockholm Female Coronary Risk (FemCorRisk) study of community women with CAD, marital stress predicted a poor prognosis with three times the increased risk of coronary events after controlling for other risk factors [155]. In 300 young and middle-aged patients (ages 18 to 60 years) with a history of a recent MI, chronic stress burden with self-reported early-life trauma was also found to be an independent risk factor for adverse CVD outcomes in both men and women (66% AA and 50% women) [156]. In another large, prospective study of 23,196 men and women (ages 20 to 54 years), childhood adversities were more powerful predictors of CVD in women compared to men [157].

While conditions such as post-traumatic stress disorder are being increasingly recognized as risk factors for CVD [158], the link between depression and its impact on heart disease is already well established [159]. Depression is associated with autonomic nervous system dysregulation as well as inflammation [152, 160]. Patients who are depressed are also less likely to make heart-healthy choices, have uncontrolled cardiac risk factors, lower medication compliance, and medical follow-up. In the Women’s Health Initiative (WHI), depression was an independent predictor of cardiovascular death [161]; similarly, in the Nurses’ Health Study, depression was associated with an increased incidence of adverse cardiac events [162].

Several studies have looked at how neurobiological mechanisms and emotions may influence stress physiology and lead to heart disease [154, 163]. In patients with stable CAD, even transient endothelial dysfunction, measured by flow-mediated dilation of the brachial artery during mental stress tasks, was a prognostic marker associated with adverse outcomes (composite endpoint of CV death, MI, revascularization, and health failure hospitalization) after adjusting for medical history and sociodemographic risk factors [164]. Women, in particular, seem to be more at risk for adverse consequences of mental stress. For example, young women with a history of an MI have more ischemia with mental stress compared to young men with an MI [165, 166]. Among 918 patients with stable CAD, those with mental stress ischemia (16% of the cohort) had increased cardiovascular death or non-fatal MI compared to those with no mental-stress ischemia at a median of 5 years of follow-up. Furthermore, compared to young men, young women with CAD had a greater inflammatory response with interleukin-6 to mental stress [167]. Sex-differences in vascular reactivity to mental stress have also been documented, with women having more microvascular vasoconstriction in response to laboratory-induced mental stress. Similarly, post-menopausal women are more susceptible to takotsubo syndrome after a significant emotional or physical stressor [168].

### 3.5 Anti-Neoplastic Treatments and Cardiotoxicity

**Fig. 2.**
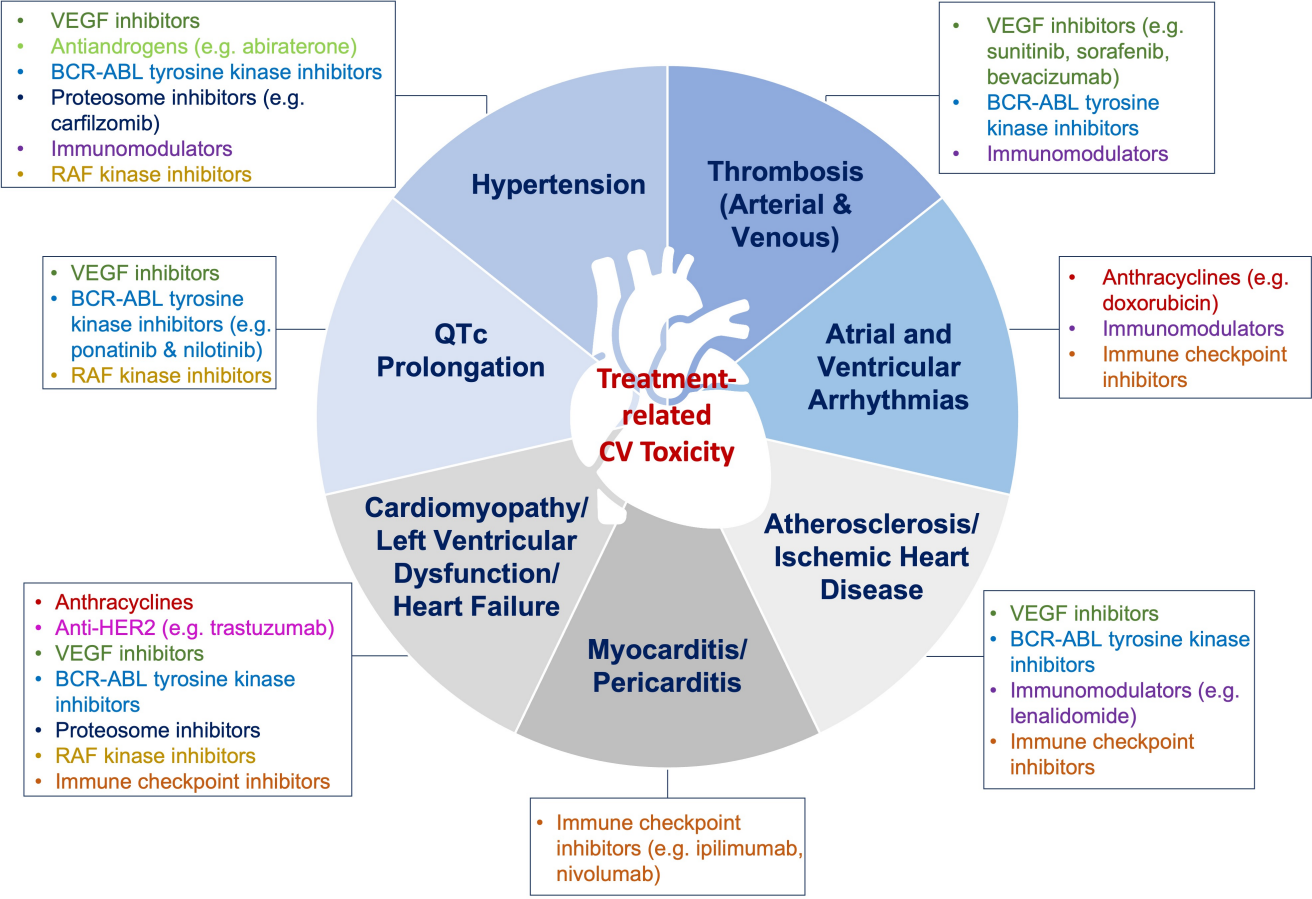
**Cardiotoxic effects of chemotherapy**. Chemotherapeutic agents are associated with various cardiotoxic effects. Timely cardiovascular assessment prior to chemotherapy initiation, and close monitoring, as well as surveillance post-therapy, are recommended. VEGF, Vascular endothelial growth factor.

While anti-neoplastic therapy is necessary to combat cancer, some types are associated with cardio-toxic effects and increase CVD risk. Over the past decade the field of cardio-oncology has emerged to address consequences of cancer therapy not only in the short-term, but also long-term in cancer survivors. A consensus statement defining cardiovascular toxicities from cancer was recently published by the International Cardio-Oncology Society (IC-OS) [169]. Anthracycline-based therapy has been known to be associated with cardiomyopathy, and newer agents such as tyrosine kinase inhibitors that target vascular endothelial growth factor (VEGF) (e.g., bevacizumab, sunitinib, sorafenib); are associated with HTN and left ventricular dysfunction (Fig. 2) [169, 170]. Baseline cardiovascular risk assessment in patients who are undergoing chemotherapy can be helpful; since as the number of CVD risk factors increase, the risk of cardiotoxicity also increases [171]. A comprehensive position statement on risk assessment tools to address baseline risk in patients who are starting chemotherapy has been recently published by the European Society of Cardiology and ICOS [171]. While a comprehensive review of cardiotoxic effects related to specific cancers and chemotherapy is beyond the scope of this manuscript, we have focused on breast cancer since it is the most common cancer in women.

While advances in early detection and targeted treatment have led to declining death rates in breast cancer, some therapies have been associated with significant adverse cardiac events [172, 173, 174, 175, 176]. There are an estimated 3.8 million breast cancer survivors in the U.S. [177]. Adverse events, including myocardial ischemia, heart failure, venous thromboembolism, and bradycardia may result from breast cancer treatment, among which anthracyclines carry the highest risk of cardiotoxicity [178]. Following long-term therapy, a large percentage of patients exposed to anthracycline-based therapy develop abnormalities in cardiac structure and function, placing them at five-times the risk of heart failure compared to patients treated with non-anthracycline-containing chemotherapy [173]. Women who have a higher baseline burden of CVD risk factors are particularly vulnerable to adverse consequences following cancer chemotherapy [179]. The cardiotoxic effects of doxorubicin are dose-dependent, with heart failure noted in 26% of patients given the maximum lifetime cumulative dose of 550 mg/$m^{2}$ [178]. The myocardial cellular damage that leads to direct myocardial injury and myocyte apoptosis is thought to be irreversible (Type I cardiotoxicity). Therefore, anthracycline-based agents are associated with significant mortality and morbidity [180, 181]. Other groups of chemotherapy agents (e.g., trastuzumab) generally do not cause direct myocellular damage and are associated with reversible left ventricular dysfunction (Type II cardiotoxicity) [182]. A retrospective cohort study of 12,500 women with breast cancer demonstrated a hazard ratio (HR) of 1.4 in patients on anthracycline alone, a HR of 4.1 in patients treated with trastuzumab alone, and a HR of 7.19 in those treated with both agents as compared to patients who received no chemotherapy, indicating a higher risk when anthracycline and trastuzumab are combined [183]. Other treatments associated with LV dysfunction and heart failure include alkylating agents (cyclophosphamides), anti-microtubule agents (taxanes), and monoclonal antibodies (trastuzumab). Cardiac ischemia has been noted in approximately 5% of patients treated with taxanes [184]. Alkylating agents can cause mild cardiac arrhythmias [185]. Anti-microtubule agents may cause myocardial ischemia, heart failure, ventricular tachycardia, and atrioventricular block [186]. A recent meta-analysis showed that several medications, including statins, were effective in reducing the risk of cardiac injury in cancer patients [173]. Cardiovascular toxicity resulting from radiotherapy, predominately for left-sided breast cancer, has been well-documented, especially among women receiving whole heart doses of ≥5 gray, as was the previous standard of care [187]. Radiation-related injury may include structural damage to the heart, such as fibrosis, pericardial adhesions, microvascular damage and valvular stenosis, as well as damage to the coronary arteries [188].

Chest wall or mediastinal radiation for treatment of malignancies such as Hodgkin’s lymphoma and breast cancer is associated with coronary atherosclerosis, as well as pericardial and valvular disease. The risk of radiation-induced heart disease increases in the presence of risk factors. An elevated risk for CVD starts within the 5 years of radiation exposure, and the rate of events increases by 7.4% per gray of radiation [189]. Left-breast radiation doses to the heart are higher than right-sided breast radiation and is associated with atherosclerosis in both the left and right coronary arteries [190]. It is important to continue to screen for CVD risk factors and atherosclerosis to guide the management in patients exposed to cardio-toxic chemotherapy or radiation therapy.

## 4. Additional Novel Risk Factors

Our understanding of CVD continues to expand with discoveries of new and potentially targetable risk factors. The gut microbiome is an emerging risk factor implicated in CVD. There may be sex-specific differences in intestinal dysbiosis that if corrected, could improve CVD risk, especially in women [191, 192, 193]. Gut microbiome and intestinal barrier dysfunction has been linked to HTN [193]. In a pilot study, Zonulin, a gut epithelial tight junction protein regulator, was found to be elevated in those patients with HTN compared to controls, and significantly correlated with elevated systolic blood pressure ($R^{2}$ = 0.5301, *p *< 0.0001) [194]. Arterial stiffness is a measure of vascular compliance and predictive of HTN and adverse cardiovascular outcomes [195]. A study in women showed the potential mechanistic role of gut microbiome on influencing arterial stiffness. Increased diversity in the gut microbiome was inversely correlated with arterial stiffness in women and was linked to the presence of specific microbial metabolites associated with an increased risk of CVD [196].

Another novel biomarker under investigation for early detection of arterial stiffness and HTN is marinobufagenin (MBG) [197]. This is a ${\mathrm{Na}}^{+}$$K^{+}$-ATPase inhibitor endogenously released in response to NaCl ingestion. MBG inhibits renal reabsorption of sodium at the proximal tubule. This increases urinary excretion of sodium, and results in vasoconstriction, and increases systolic blood pressure [197, 198]. To understand the role of MBG in salt-sensitive HTN and arterial stiffness, middle-aged adults (n = 11) with moderate systolic blood pressure were placed on dietary sodium restrictions. Collected urinary samples had decreased concentrations of MBG and these individuals demonstrated reduced systolic blood pressure and aortic stiffness [198]. In a similar study conducted in young adults, the African Prospective study on the Early Detection and Identification of Cardiovascular disease and Hypertension (African-PREDICT), higher MBG levels at a young age was also found to contribute to increased artery stiffness and adverse hemodynamics [199]. Another study showed a positive correlation between MBG/${\mathrm{Na}}^{+}$ excretion ratios and central and systolic blood pressures, especially in young black women [200].

### Knowledge Gaps

There is a substantial lack of awareness, especially among young and racial/ethnic minority women, that CVD is a major health threat in women, and targeted educational efforts are needed [201]. Multi-specialty and team-based care to improve CVD risk factor control is needed to make progress in the battle against CVD in women [202]. Moreover, an improved understanding of how to practically incorporate socio-cultural factors and social determinants to guide care in women is essential [1, 203]. Several questions regarding optimal risk factor assessment and deployment of pharmacotherapy remain: Does early treatment of cardiac risk factors in women with adverse pregnancy outcomes lead to a significant reduction in mortality? Should preventive strategies such as statins and aspirin be recommended to all patients with underlying chronic autoimmune inflammatory disorders? Should young women with autoimmune inflammatory disorders or adverse pregnancy outcomes undergo coronary artery calcium scoring to help guide primary prevention, and if so, starting at what age? Does early initiation of menopause hormone therapy, especially transdermal formulations, during the transition to menopause in younger women under the age of 60, attenuate the cardiometabolic and hypertensive risk associated with declining estrogen levels?

Adverse drug reactions (ADRs) can range from mild, simple rash, to severe and life-threatening conditions such as angioedema. There has been a historic lack of comparisons of the rate of adverse reactions between men and women in clinical trials [204]. There are sex-specific differences in the incidence of drug reactions among common cardiovascular medications, with women experiencing adverse events more often than men [205]. For example, an increased incidence of ADRs has been observed with ACE-I (enalapril) (odds ratio 1.30) and particularly a higher risk of cough in women compared to men (odds ratio: 2.38) [206]. In a meta-analysis comparing the effects of statins on cardiovascular outcomes, no differences in adverse drug reactions were found in women compared to men; however, data for myopathy and new-onset diabetes were limited [207].

**Fig. 3.**
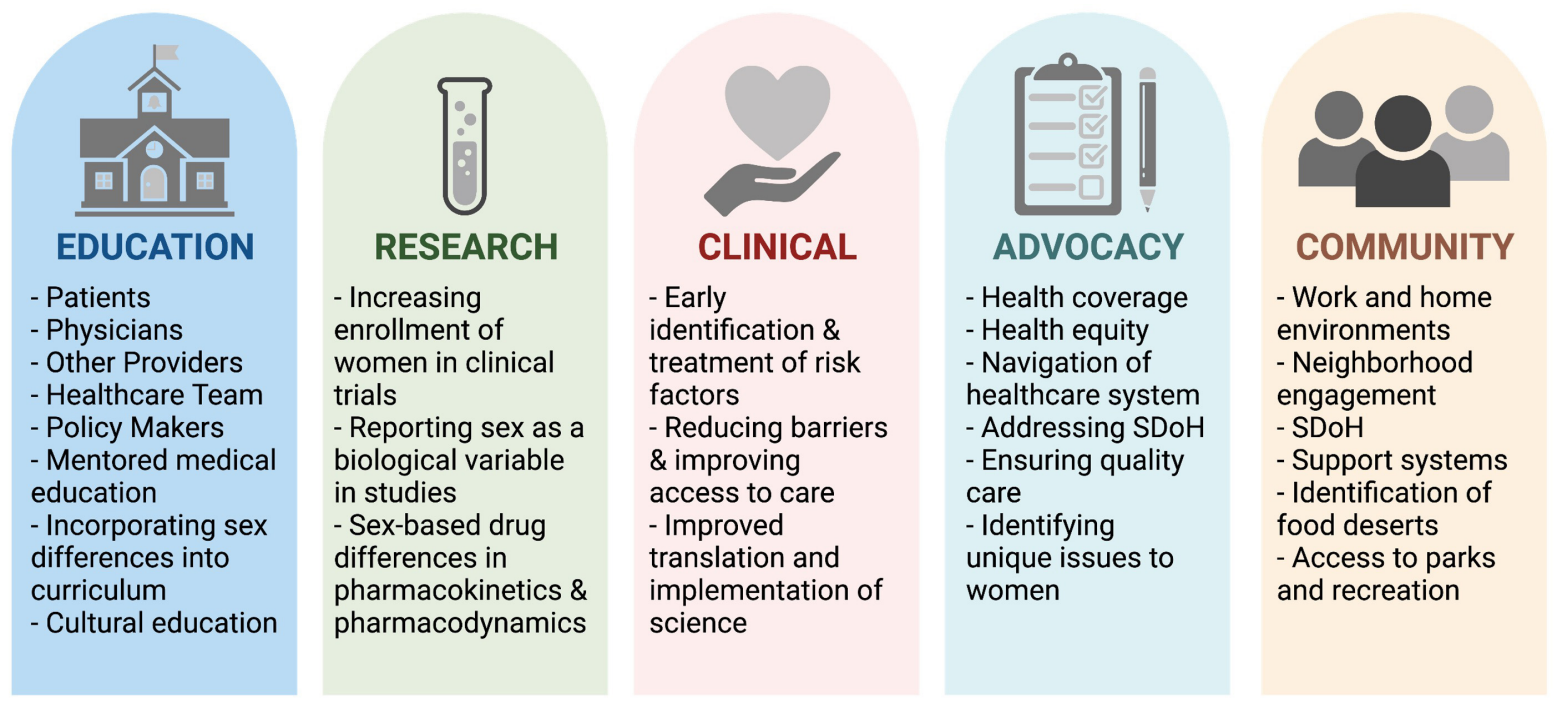
**Pillars for integrated cardiovascular risk factor care in women**. Optimizing and improving CVD care for women requires efforts at all levels. SDoH, Social Determinants of Health. Figure created using https://Biorender.com.

What are the main barriers that prevent optimal CVD risk assessment care in young women and what system level changes can be optimized to reduce the sex-based disparities in CVD care? Questions related to pathobiological differences in CVD in women vs. men should also continue to be investigated [208]. Improved education, screening, identification, and treatment (pharmacologic and lifestyle-based approaches) of CVD risk factors in women require engagement across all levels and a systems-based approach (Fig. 3) [209].

## 5. Conclusions 

In addition to traditional CVD risk factors, unique sex-specific risk factors contribute to increased CVD morbidity and mortality in women. Complications of pregnancy, autoimmune/inflammatory conditions, premature menopause, and stress/depression are some of the nontraditional risk factors that may help identify women at increased CVD risk. Women have a high burden of comorbid conditions that impact their health-related quality of life. Early identification and treatment of modifiable CVD risk factors may alleviate CVD risk in women. A multi-disciplinary approach to not only provide comprehensive care, but also targeted public health education regarding CVD risk, will be beneficial to all women.
